# Supplementary material for: The Agreement between Radiography and Fluoroscopy as Diagnostic Tools for Tracheal Collapse in Dogs
Source: Animals (Basel). 2023 Apr 22;13(9):1434. doi: 10.3390/ani13091434 (PMC10177530; doi:10.3390/ani13091434)
Supplement: Supplementary file 1 [file animals-13-01434-s001.zip › animals-2306106-supplementary.pdf]

Supplementary Table S1. Results of agreement test between fluoroscopy and radiography were reported with kappa statistic .

	Regions	Agreement (%)	Both Present	Both Absent	Flu+/Rad-	Flu-/Rad+	P Value of binomial distribution	Kappa	Odds Ratio (CI)
				Cutoff at 16.4%					
Day 0	Cervical	23/29 (79.31)	4/29	19/29	0/29	6/29	0.031	0.47	n/a
	Thoracic inlet	14/29 (48.28)	5/29	9/29	7/29	8/29	1.000	−0.05	1.1 (0.36–3.70)
	Intra thoracic	14/29 (48.28)	3/29	11/29	10/29	5/29	0.302	−0.09	0.5 (0.13–1.61)
	Carina	13/29 (44.83)	8/29	5/29	12/29	4/29	0.077	−0.04	0.3 (0.08–1.10)
Day 14	Cervical	21/29 (72.41)	2/29	19/29	3/29	5/29	0.727	0.17	1.7 (0.32–10.73)
	Thoracic inlet	17/29 (58.32)	2/29	15/29	6/29	6/29	1.000	−0.04	1.0 (0.27–3.74)
	Intra thoracic	14/29 (48.28)	4/29	10/29	7/29	8/29	1.000	−0.08	1.1 (0.36–3.70)
	Carina	14/29 (48.28)	5/29	9/29	12/29	3/29	0.035	0.04	0.3 (0.05–0.93)
Day 56	Cervical	21/29 (72.41)	0/29	21/29	2/29	6/29	0.289	−0.12	3.0 (0.54–30.39)
	Thoracic inlet	17/29 (58.62)	3/29	14/29	4/29	8/29	0.388	0.05	2.0 (0.54–9.08)
	Intra thoracic	13/29 (44.83)	0/29	13/29	11/29	5/29	0.210	−0.31	0.5 (0.12–1.42)
	Carina	13/29 (44.83)	6/29	7/29	11/29	5/29	0.210	−0.06	0.5 (0.12–1.42)
				Cutoff at 10%					
Day 0	Cervical	17/29 (58.6)	3/29	8/29	6/29	6/29	1.000	0.17	1.0 (0.27–3.74)
	Thoracic inlet	18/29 (62.0)	15/29	3/29	8/29	3/29	0.227	0.12	0.4 (0.06–1.56)
	Intra thoracic	20/29 (69)	16/29	4/29	8/29	1/29	0.039	0.30	0.1 (0.00–0.93)
	Carina	20/29 (68.9)	19/29	1/29	8/29	1/29	0.039	0.08	0.1 (0.00–0.93)
Day 14	Cervical	14/29 (48.3)	4/29	10/29	7/29	8/29	1.000	−0.08	1.1 (0.36–3.70)
	Thoracic inlet	16/29 (55.2)	8/29	8/29	6/29	7/29	1.000	0.11	1.2 (0.33–4.20)
	Intra thoracic	15/29 (51.7)	14/29	1/29	5/29	9/29	0.424	−0.18	1.8 (0.54–6.83)
	Carina	17/29 (58.6)	14/29	3/29	11/29	1/29	0.006	0.15	0.1 (0.00–0.63)
Day 56	Cervical	18/29 (60)	6/29	12/29	3/29	9/29	0.146	0.20	3.0 (0.75–17.23)
	Thoracic inlet	11/29 (36.7)	6/29	5/29	10/29	9/29	1.000	−0.27	0.9 (0.32–2.46)
	Intra thoracic	13/29 (46.7)	9/29	5/29	12/29	4/29	0.077	−0.01	0.33 (0.08–1.10)
	Carina	18/29 (62.0)	16/29	2/29	11/29	1/29	0.006	0.10	0.1 (0.00–0.63)

*Binomial distribution was be calculated due to discordant paired less than 25

Supplementary Table S2. Treatment using medication per oral route, nebulization route, and supplement for manage tracheal collapse disease in dogs.

Treatment	Before visit	Day 0	Day 14	Day 28	Day 56
Per oral					
Doxophylline	1	1	0	0	0
Theophylline	1	0	0	0	0
Aminophylline	3	3	0	0	0
Carbocysteine	7	5	2	1	2
Acetylcysteine	1	1	1	1	0
Codeine	0	0	0	0	0
Prednisolone	4	1	0	0	0
Hydroxyzine	1	0	0	0	0
Amoxycillin	1	0	0	0	0
Nebulization					
NSS	2	3	3	2	2
Salbutamol	3	4	1	2	2
Fluticasone	2	1	0	0	0
Budesonide	2	2	2	2	2
Supplement					
Omega-3	0	15	15	15	15

Supplementary Table S3. Cough symptom evaluation questionnaire (modified from Jeung et al.) [1].

Variables of cough	Criteria Score		Clinical Correlate
Duration	□	1	Less than a minute
	□	2	1–5 min
	□	3	5–10 min
	□	4	10–20 min
	□	5	Over 20 min
	□	6	Over an hour
Interval	□	1	Very rare (in weeks)
	□	2	Only excitement (in days)
	□	3	Over 24 h
	□	4	Every 8–12 h
	□	5	Every 6–8 h
	□	6	Every 4–6 h
	□	7	Every 2–4 h
	□	8	Every 1–2 h

Reference

1. Jeung, S.Y.; Sohn, S.J.; An, J.H.; Chae, H.K.; Li, Q.; Choi, M.; Yoon, J.; Song, W.J.; Youn, H.Y. A retrospective study of theophylline-based therapy with tracheal collapse in small-breed dogs: 47 cases (2013–2017). *J Vet Sci*. **2019**, *20(5)*, e57. doi:10.4142/jvs.2019.20.e57

Supplementary Table S4. Results of cough symptom evaluation questionnaire (modified from Jeung et al.) [1].

Variables	Day 0	Day 14	Day 28	Day 56

Cough duration	1(1–2)	1(1–2)	1(1–2)	1(1–2)
Cough interval	3(1–5) [ab]	2(1–3.5) [a]	2(1–2) [b]	2(1–2)

Distribution of Cough symptom evaluation questionnaire outcome of 29 dogs. Results are shown as median with interquartile ranges. Comparisons between visits within subjects each group were tested by Friedman with pairwise Wilcoxon signed-rank test. The values with same superscript letters in a column of each group are significantly different ($p < 0.05$; [a], $p = 0.003$, [b], $p = 0.001$).

Reference

1. Jeung, S.Y.; Sohn, S.J.; An, J.H.; Chae, H.K.; Li, Q.; Choi, M.; Yoon, J.; Song, W.J.; Youn, H.Y. A retrospective study of theophylline-based therapy with tracheal collapse in small-breed dogs: 47 cases (2013–2017). *J Vet Sci*. **2019**, *20(5)*, e57. doi:10.4142/jvs.2019.20.e57
